# Therapeutic potential of bioactive compounds from Punica granatum extracts against aging and complicity of FOXO orthologue DAF-16 in *Caenorhabditis elegans*

**DOI:** 10.17179/excli2020-3011

**Published:** 2021-01-11

**Authors:** Mukesh G. Chaubey, Anita P. Chauhan, Pooja R. Chokshi, Rahi S. Amin, Stuti N. Patel, Datta Madamwar, Rajesh P. Rastogi, Niraj Kumar Singh

**Affiliations:** 1 Department of Biotechnology, Shri Alpesh N. Patel P.G. Institute of Science and Research, Anand-388001, Gujarat, India; 2 Post-Graduate Department of Biosciences, UGC-Centre of Advanced Study, Sardar Patel University, Vadtal Road, Satellite Campus, Bakrol, Anand, Gujarat 388 315, India; 3 P. D. Patel Institute of Applied Sciences, Charotar University of Science and Technology, CHARUSAT campus, Changa 388421, Anand, Gujarat, India; 4 Ministry of Environment, Forest and Climate Change, Indira Paryavaran Bhawan, New Delhi 110003, India

**Keywords:** Punica granatum, C. elegans, antioxidants, anti-aging, free radicals, oxidative stress

## Abstract

Some natural fruits have significant importance in improving health which provides many nutritional supplements essential to maintain proper metabolism with the age. In this study, phytochemical screening of extract (methanolic) of *Punica granatum* arils, outer and inner peels was confirmed by the respective spot tests. Quantification of phytochemical constituents revealed the plentiful of total phenols in the outer peels in comparison to inner peels and juice whereas total flavonoids and vitamin C are abundant in inner peel and juice, respectively. High-performance liquid chromatography, Gas chromatography along with mass spectrometry and Fourier-transform infrared spectroscopy analysis revealed the presence of compound 9, 17-octadecadienal, (Z) in the outer/inner peels. A compound N-hexadecanoic acid was also observed in the outer peels. Extracts from every section of the fruits were comprehensively evaluated for their antioxidant activity. Contrary to fruit aril juice, the extracts of outer and inner peels exhibited significant and dose-dependent *in vitro* antioxidant and radical-scavenging potentials. The supplementation of *P. granatum* extracts (PGEs) significantly enhanced the lifespan of *C. elegans*. The protective effect of PGEs was also observed against oxidative stress in *C. elegans*. Additionally, the involvement of FOXO orthologue DAF-16 dependent longevity was obtained with PGEs (outer peel and inner peel) fed TJ356 worms. Overall, the results indicate the vital role of PGEs especially the extracts of outer peels in life-saving mechanisms of *C. elegans* by virtue of their antioxidant asset and life-prolonging effects via *daf-16* dependent Insulin signaling pathway. See also Figure 1(Fig. 1).

## Introduction

Several epidemiological reports have revealed that daily intake of green vegetables and fruits acts as therapeutic agents for reducing the possibility of age-related chronic diseases. Both genetic and environmental signals influence aging (Kenyon, 2010[29]). The free radical theory as well as the mitochondrial theory of aging has proposed that the occurrence of aging is due to cellular accumulation of free radical damage over time (Hekimi et al., 2011[23]). Intracellular damage caused by free radical or oxidative stress leads to different types of diseases such as cancer, arthritis, atherosclerosis, Alzheimer's disease, and diabetes (Clancy and Birdsall, 2013[16]; Sonani et al., 2014[43]). The uncontrolled output of free radicals is associated with lipid peroxidation that results in cellular damage, tissue injury, or gene mutation (Negi and Jayaprakasha, 2003[34]). The natural antioxidant compounds are found in many vegetables and fruits (Omenn et al., 1996[35]). It has been established that endogenous antioxidants enhance the cellular ability to protect vital biological functions against oxidative stress (Chaubey et al., 2019[13]). Plenty of antioxidants in some fruits and vegetables are shown to be critically important in reducing the risk of degenerative diseases and neurological diseases (Ames et al., 1993[3]; Sonani et al., 2014[43]).

The pomegranate (*Punica granatum *L) is recognized as an ancient edible fruit and is considered as **“**Food of Gods**” **and as it is symbolic of abundance, productiveness, and well-being. The pomegranate plant is a deciduous shrub with about 5 to 8-meter height (Bhandary et al., 2012[6]). It is widely found in tropical and subtropical regions such as the Middle East and India, and it has been used since ancient times due to its medicinal properties (Wang et al., 2011[47]). Recent interest in using *P. granatum *is because of its anti-parasitic, anti-microbial, anti-carcinogenic, anti-oxidant, anti-inflammatory, anti-virus, and anti-proliferative activity along with several other health benefits. These beneficial effects are attributed to the anti-oxidative properties of pomegranate fruit's phenolic compounds, tannins, and anthocyanins as well as other phytochemicals (Akpinar-Bayizit et al., 2012[1]). As the total phenolic compounds in the *P. granatum *are higher, they contribute better antioxidant activity than that of ascorbic acid (Connor et al., 2002[17]; Kalt et al., 1999[26]; Kang and Saltveit, 2002[27]). To check the anti-aging property of *P. granatum *extracts, a eukaryotic nematode *Caenorhabditis elegans* was used as a model organism for undertaking the present study.

*C. elegans *is an expository and suitable model organism for *in vivo* studies (Brenner, 1974[11]; Kenyon, 2010[29]). The physiological and behavioral decline in mammals is the physiological markers of aging. About 80 % of *C. elegans *genes and proteins are homologous to human genes and they also involve the genes implicated in aging and similar physiological decline occurred in mammals (Bell et al., 2009[4]; Braeckman and Vanfleteren, 2007[9]). The aging development in *C. elegans *is almost identical to humans in many characteristics; for instance, reduced motility, dropped neuronal functions, immunosenescence, declined rate of reproduction, and elevated deposition of harmful metabolites such as lipofuscin (Tissenbaum, 2015[46])*.* Therefore, *C. elegans* is believed as a suitable model to check the effects of therapeutic compounds which may restrict aging. Use of *P. granatum* outer peel and inner peel have shown identical nutraceutical food supplement activity as compared to *P. granatum* extracts (PGEs) aril juice. The anti-cancer characteristics of pomegranate extract are because of their ability to act in the cell cycle, proliferation, invasion, and angiogenesis (Chaves et al., 2020[14]). Since different self-protective mechanisms of *C. elegans *via either downregulation of DAF-2-AGE-1 (Ageing alteration-1)-DAF-16 (Abnormal dauer formation-16) insulin signaling pathway (Henis-Korenblit et al., 2010[24]; Kenyon, 2010[29]; Sonani et al., 2014[42]) or reactive oxygen species we focused to trace the effect of PGEs concerning *DAF-16*.

The extracts of arils, outer peels, and inner peels may have high therapeutic importance in overcoming the oxidative stress-induced age-related complications that are very common due to changed lifestyle in the current times. In the present study, phytochemical contents of *P. granatum* were chemically characterized and investigated for their anti-oxidant and anti-aging functions, and the mechanism of PGEs with the potent complicity of *DAF-16* was also determined.

## Materials and Methods

### Sample preparation and extraction

Fresh *P. granatum* fruits were collected from the local vegetable market of Anand town in Gujarat, India. The fruits were properly washed, outer/inner peels were removed manually, and freeze-dried in liquid nitrogen before storing at -80 °C. The frozen peels were lyophilized at -100 °C in a freeze-drier (Sentry 2.0, VirTis, SP Scientific), grounded into a fine powder, and kept in an air-tight container at -20 °C until further use. The seeds with surrounding arils were discreted from mesocarp. The fresh juice was extracted by squeezing and obtained juice was stored in dark conditions at 4 °C.

The functional products from powdered outer/inner peels were extracted using 75 % methanol (1:5 w/v; HPLC grade). The supernatant was obtained from homogenate by centrifugation (10,000 × g, 4 °C, 5 min) and, further, the supernatant was evaporated in a vacuum evaporator (SPD111V, Thermo Electron Corp.). The remaining residue was re-dissolved in either MQ water (Juice) or 75 % methanol (outer peel and inner peel) and subjected to further analysis.

### Spot test 

Various spot tests were executed to qualitatively deduce the existence of different chemical components such as alkaloids, flavonoids, tannins, and vitamin C in extracts of all parts of *P. granatum* fruits (Bhandary et al., 2012[6]).

### Quantitative estimation 

#### Estimation of total phenols and flavonoids

Total phenolic contents of *P. granatum* outer peel, inner peel, and aril juice were analyzed by Folin-Ciocalteu reagent (FCR) with slight modifications (Singleton et al. 1999[40]). The reaction mixture was kept at room temperature for 90 minutes and the absorption spectrum of the test and the standard solution was measured at 760 nm using a UV-Visible spectrophotometer (Specord 210, Analytik Jena AG, Jena, Germany). Overall phenolic constituents were expressed as tannic acid equivalent in µg/g of extract.

The total flavonoid constituents were assessed by colorimetric method as described by Zhishen et al. (1999[54]). The total flavonoid constituent in the test samples was analyzed by measuring the O.D (absorbance) at 510 nm of the reaction mixture and exhibited as mg quercetin equivalents/g of extract.

#### Estimation of ascorbic acid 

The appearance of Vitamin C in the outer peel, inner peel, and aril was estimated by a colorimetric method using 2, 4-dinitrophenylhdrazine (2, 4-DNPH) (Strohecker and Henning, 1967[44]). The standard solution of ascorbic acid was also measured in the same manner as described in the method (10 to 100 µg/ml). Vitamin C was expressed as mg of ascorbic acid equivalent/g of extract.

### GC-MS (Gas chromatography-mass spectroscopy) analysis

The GC/MS analysis of *P. granatum *(methanolic extract) was executed by a Perkin Elmer Clarus® 500 Gas Chromatograph equipped with a built-in syringe auto-sampler (AOC-20i) and a mass spectrometer interfaced with gas chromatography comprising an Elite-5MS capillary column (30 m x 0.25 mm, 0.25 µm). Helium (1 ml/min) was used as a carrier gas for the separation of components. The sample extract (1 µl) was injected through an auto-injector. During the GC extraction process, the parameter set for the oven temperature was 80 °C to 280 °C with a gradual increase of 10 °C/min. The injector temperature was set at 220 ºC. The mass spectrum was taken at 70 eV ionization energy with fragments scanned at 20 to 400 *m/z*. The identification of the different phyto-compounds was done by GC by comparing their relative retention time (RRt) values and their MS fragments were identified employing the National Institute of Standard and Technology (NIST) MS database (version 2.0) library.

### Fourier Transform Infrared Spectroscopy (FTIR) analysis

Perkin Elmer FTIR spectrometer (Spectrum GX, PerkinElmer, USA) was used to record the IR spectra. The freeze-dried fine powdery test sample (2 mg) was added with potassium bromide (KBr: 200 mg) and analyzed at mid-IR region (400-4000 cm^−1^) with resolution of 1 cm^-1 ^against the blank of KBr (100 %). All the spectra were collected in three replicates (n=3).

### In vitro antioxidant activity

The antioxidant activity was analyzed by different assay such as 2,2-diphenyl-1-picryl-hydrazyl (DPPH) scavenging assay, ferric ion reducing antioxidant power (FRAP) assay, Hydrogen peroxide (H_2_O_2_) scavenging assay, and reducing power (RP) assay. 

#### DPPH free-radical scavenging assay

The free-radical scavenging capacity of methanolic extract was analyzed by using a method as described earlier (Brand-Williams et al., 1995[10]). The absorbance of each sample was measured against methanol without DPPH and results were expressed as percent inhibition of DPPH radical.

DPPH scavenging effect (%) = A_0 _- A_1 _/ A_1_ × 100

A_0 = _absorbance of control reaction; A_1 _= absorbance in presence of sample.

#### FRAP assay

This assay was performed as described earlier (Benzie and Strain, 1996[5]). Concisely, 100 µl of methanolic extract was treated with 2600 µl of freshly prepared FRAP (Ferric reducing ability of plasma) reagent and 300 µl of distilled water. The resultant mixture was kept at 37 °C for 30 minutes in the dark and absorbance at 593 nm was recorded against a blank sample. 

#### H_2_O_2 _scavenging assay

The potential of *P. granatum* extracts to remove hydrogen peroxide was evaluated according to the earlier described method (Ruch et al., 1989[39]). The phosphate buffer, without the H_2_O_2_ solution, was taken as a blank sample. The percent scavenging of H_2_O_2_ was evaluated using the following formula

% H_2_O_2_ scavenging = [(A_C _- A_S_) / A_C_] × 100

where A_C _=absorbance of control, A_S_ = absorbance of the sample.

#### Reducing power assay 

The reducing power of *P. granatum* extracts was verified as reported earlier (Oyaızu, 1986[36]). After 10 min of reaction time, absorbance at 700 nm was recorded and the percentage of reducing power of each sample was measured using the following formula.

% Increase in reducing power = [(Absorbance of sample / absorbance of blank) - 1] × 100.

### Caenorhabditis elegans: cultivation and synchronization 

The wild type N2 Bristol strain and TJ356 strain of *C. elegans *were taken for investigating the effect of PGEs. These strains were obtained from the Caenorhabditis Genetic Centre (CGC), University of Minnesota, Minneapolis MN. The cultivation of strains was executed at 20 °C on nematode growth medium (NGM) agar plates seeded with the *E. coli* OP50 strain as a food source (Brenner, 1974[11]). To obtain the age-synchronized worms, gravid worms were treated with sodium hypochlorite solution (1 N NaOH + 5 % NaOCl) which leads to the release of the eggs from the worms. The laid out eggs were washed repeatedly in M9 buffer (3 g of KH_2_PO_4_, 6 g of Na_2_HPO_4_, 5 g of NaCl, 1 ml of 1 M MgSO_4_, prepared up to the volume of 1 L using H_2_O) to neutralize the effect of hypochlorite treatment. Subsequently, the eggs were allowed to hatch overnight at 20 °C. The obtained synchronized L1-stage worms were kept on standard NGM plates with *E. coli* OP50 as a food source and then kept at 20 °C till the L4 larval stage.

#### Lifespan assays

The obtained L4 stage worms were kept on fresh NGM agar plates and the lifespan assay was performed in triplicates at 20 °C and 25 °C with the different concentrations (10-40 μg/ml) of the fruit extracts. The worms were transferred into a fresh food plate every alternate day and the number of live and dead worms was scored. The physiological reaction to mechanical stimulus given by a platinum pick was considered as the characteristics to record dead ones (Kenyon et al., 1993[28]).

#### Oxidative stress resistance assay 

The age synchronized young N2 worms were shifted to a fresh NGM agar plate treated with equal concentration (20 μg/ml) of PGEs. The worms were grown till post adulthood and then taken for the oxidative stress assay. The worms were exposed to 10-20 mM of H_2_O_2_ solutions for 2 h (Cai et al., 2011[12]).

### DAF-16: GFP nuclear localization assay

The synchronized population of L1 stage TJ356 was grown on an NGM plate supplemented with 100 µg/ml of PGEs of outer peel and inner peel respectively, while the control plate was supplemented without PGEs extract. L4 stages worms were observed and photographs are taken (at 10 x) using a Nikkon DS-Ri2 fluorescence microscope (Eclipse Ni-E, Nikon). The fluorescence microscopy parameters were set as defaults such as filters 400/30 and 508/20 were used for excitation and emission to check the Green fluorescent protein (GFP) transgenic lines. Movement of *DAF-16 *from cytosol was observed by measuring the continuation of GFP clustering in the nuclei.

### Statistical analysis

All experiments were performed in triplicate (mean ± SD; n = 3) and statistical significance, P-value < 0.0001, was analyzed using a One-way Analysis Of Variance (ANOVA) test.

## Result and Discussion

### Qualitative and quantitative analysis of phytochemical constituents

**Figure 2:**
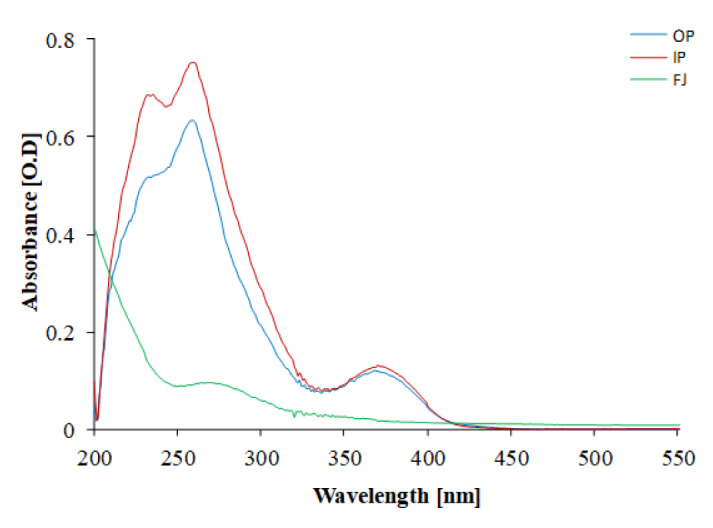
UV-visible absorption spectrum of methanolic PGEs. OP: outer peel; IP: inner peel; FJ: fruit juice extract

**Figure 3:**
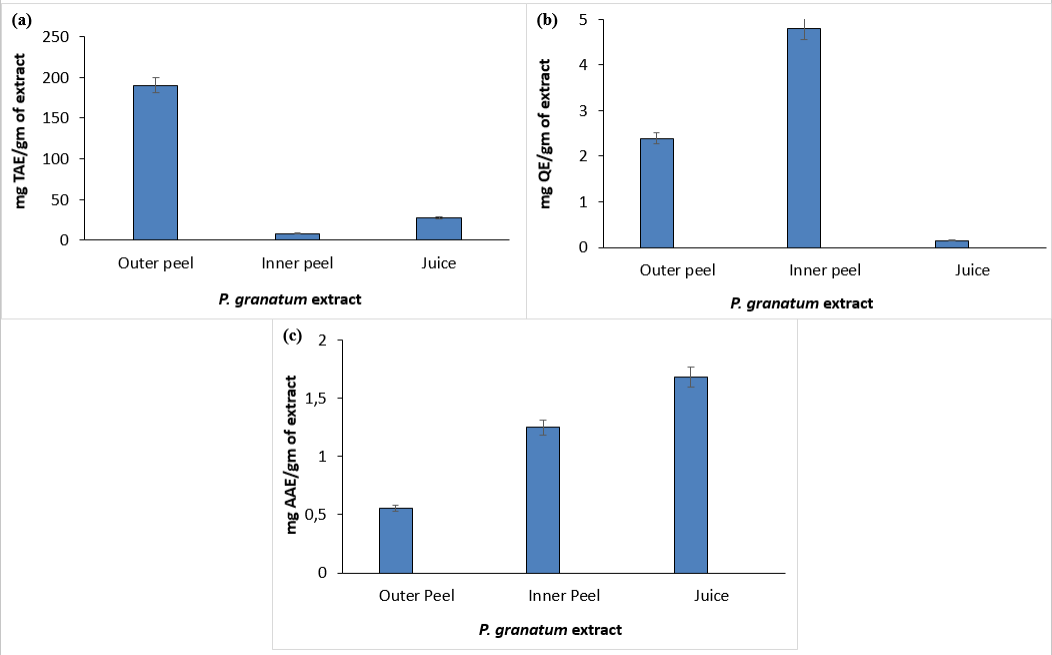
Quantitative estimation of different phytoconstituents such as total phenol (a), total flavonoids (b), and vitamin C (c) in *P. granatum*

The phytochemical assessment of PGEs was carried out to trace the existence of different chemical constituents that elicit a major pharmacological response. The presence of alkaloids, flavonoids, tannins, and vitamin C in PGEs was confirmed by various Spot tests as shown in Table 1(Tab. 1). Figure 2(Fig. 2) shows the absorption spectra of crude methanolic PGEs with a UV absorbance at around 230 ± 2 nm, 260 ± 2 nm, and maximum at 368 ± 2 nm. The quantitative analysis of *P. granatum *methanolic extracts was performed using the respective standards such as tannic acid (total phenol), ascorbic acid (vitamin C), and quercetin (total flavonoids). The high concentration of total phenol was found in the outer peel (190.55 mg/g of extract as tannic acid equivalent) of *P. granatum *compared to the inner peel (8.33 mg/g of extract as tannic acid equivalent) and juice (27.79 mg/g of extract as tannic acid equivalent) (Figure 3a(Fig. 3)) (Supplementary Table 1). While the maximum content of flavonoid was observed in the extracts of inner peel (4.80 mg/g of extract as quercetin equivalent) in comparison to outer peels (2.397 mg/g of extract as quercetin equivalent) and juice (0.154 mg/g of extract as quercetin equivalent) (Figure 3b(Fig. 3)) (Supplementary Table 2). The high content of vitamin C (1.685 mg/g of extract as ascorbic acid equivalent) was found in the juice extract as compared to the outer peel (0.555 mg/g of extract as ascorbic acid equivalent) and inner peel (1.25 mg/gm of extract as ascorbic acid equivalent) (Figure 3c(Fig. 3)) (Supplementary Table 3).

Pomegranate is a good source of several bioactive metabolites (Al-Rawahi et al., 2014[2]; Passafiume et al., 2019[37]; Young et al., 2017[48]; Zarei et al., 2011[50]; Zhang et al., 2011[52]), however, the content and chemical composition of various bioactive compounds of pomegranate depends on their cultivar, climate of the growing regions, etc. (Borochov-Neori et al., 2009[8]; Díaz-Pérez et al., 2019[19]).

#### GS-MS spectroscopy

**Figure 4:**
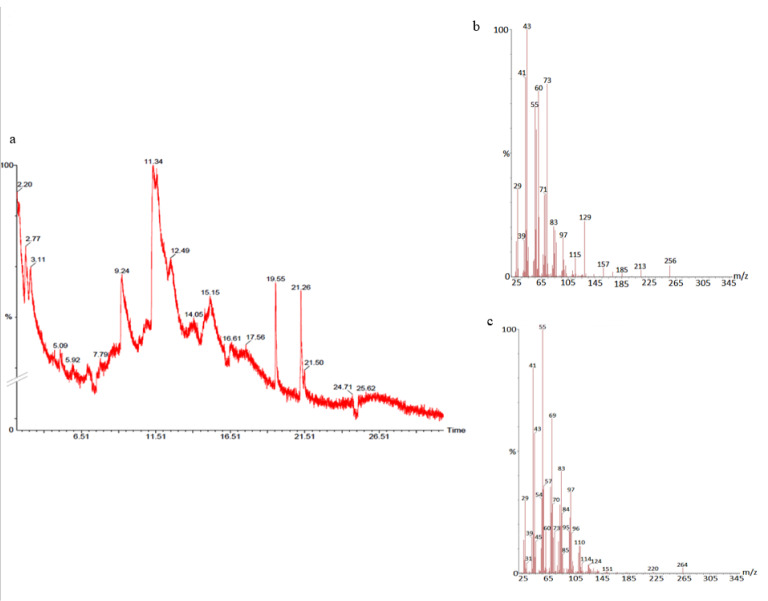
GC- chromatogram of *P. granatum* outer peel (a) and MS-spectroscopy of two distinct peaks at 19.55 min (b) and 21.26 min (c) with a prominent ion peak at *m/z* 256 (b) and 264 (c), respectively.

**Figure 5:**
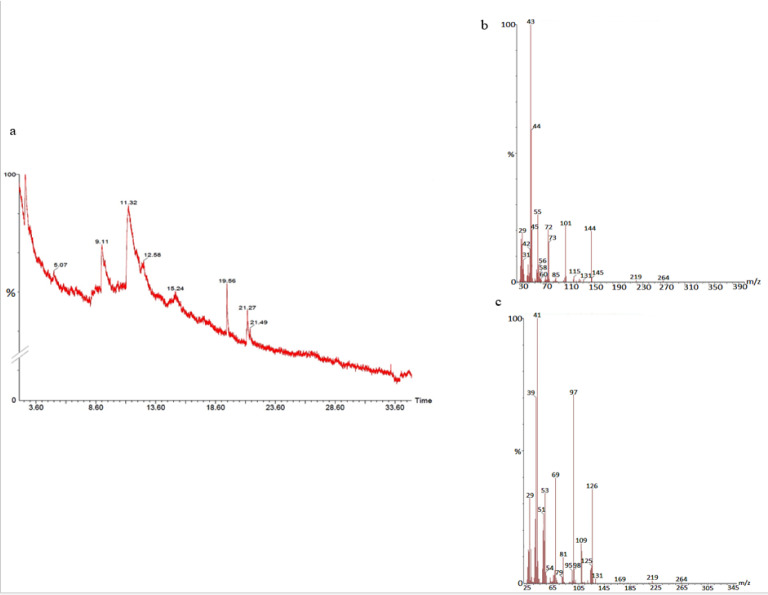
GC-chromatogram of *P. granatum* inner peel (a) and MS-spectroscopy of two distinct peaks at 9.11 min (b) and 11.32 min (c) with a common prominent ion peak at *m/z* 264.

**Figure 6:**
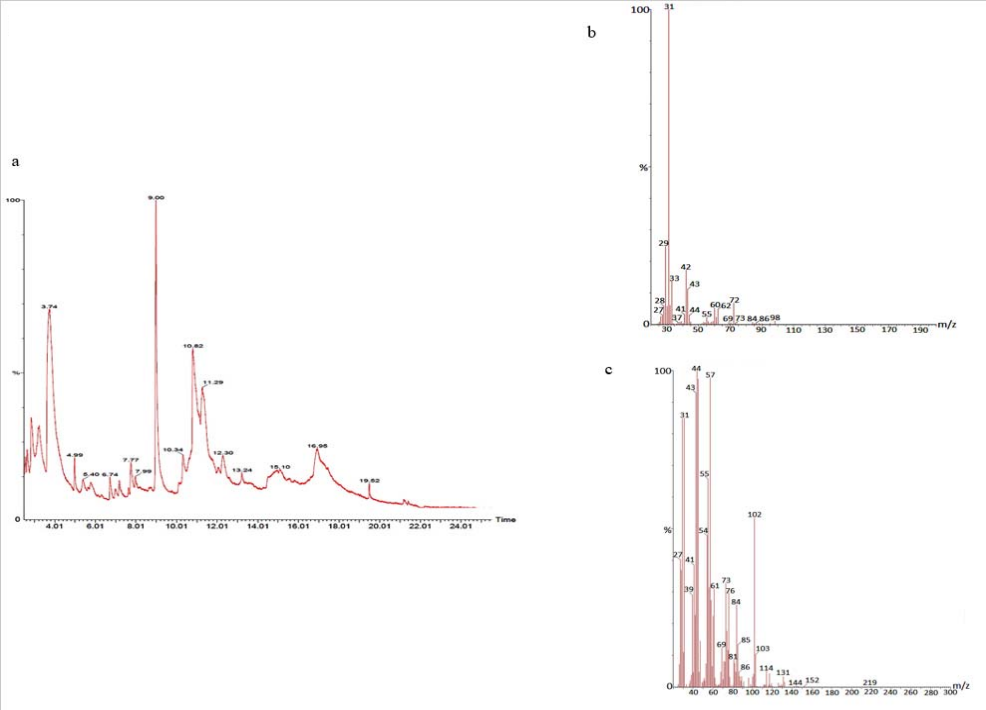
GC-chromatogram of *P. granatum* juice (a) and MS-spectroscopy of two distinct peaks at 3.73 min (b) and 16.95 min (c) with a prominent ion peak at m/z 98 (b), and 219 (c), respectively.

The GC-MS analysis of the sample revealed the presence of different compounds in the methanolic PGEs (Bonzanini et al., 2009[7]; Choi et al., 2006[15]). The percentage of the total area of peaks of the GC spectrum of some active compounds is shown in Table 2(Tab. 2). Figure 4(Fig. 4) shows the GC-MS chromatogram of methanolic extracts of outer peels. The GC chromatogram of a methanolic crude extract of outer peel showed two separate peaks at retention time (RT) 19.55 ± 1 min and 21.26 ± 1 min (Figure 4a(Fig. 4)). The prominent ion peak was obtained at *m/z* 256 (Figure 4b(Fig. 4)) and 264 (Figure 4c(Fig. 4)) denoting the appearance of N-hexadecanoic acid (palmitic acid) and anthracene 9,10-carbaldehydedioxime, respectively. Similarly, the GC spectrum of inner peels also showed two distinct peaks at 9.11 min and 11.32 min (Figure 5a(Fig. 5)) with a common prominent ion peak at *m/z *264 under MS analysis of both peaks at 9.11 min (Figure 5b(Fig. 5)) and 11.32 min (Figure 5c(Fig. 5)) indicating the presence of N-hexadecanoic acid, which is a well-known antioxidant compound. The GC chromatogram of juice extract showed two peaks at RT 3.73 min and 16.95 min (Figure 6a(Fig. 6)) correspondings to the molecular weight of 98 (Figure 6b(Fig. 6)) and 219 (Figure 6c(Fig. 6)) specifying the appearance of 6-oxabicyclo [3.1.0] hexan-3-one and S-[2-[N, N-Dimethylamino] N, N-dimethylcarbamoylthiocarbohydroximate respectively. Table 3(Tab. 3) shows the presence of some major phytoconstituents in different methanolic extracts of *P. granatum*. The GC-MS technique is a good and widely established approach to comprehend the nature of compounds that exists in different plant extracts.

#### Fourier-Transform Infrared Spectroscopy (FTIR)

**Figure 7:**
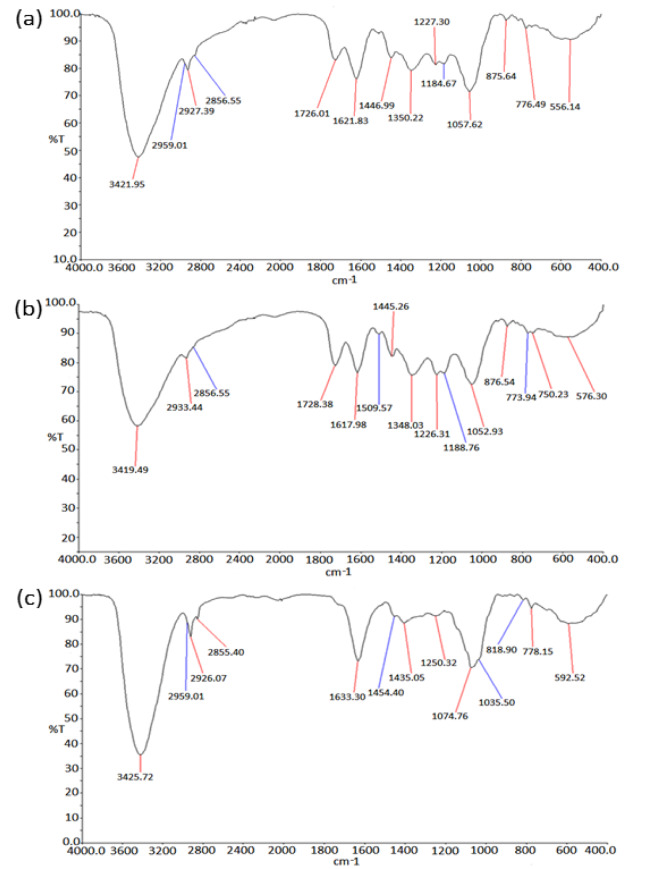
FTIR spectrum of Outer Peel (a), inner peel (b), and Juice (c)

FTIR spectroscopy technique is widely used for chemical analysis of the plant metabolites. In the present study, the FTIR spectrum of the PGEs showed major absorbance bands in each of the samples as shown in Table 4(Tab. 4). Figure 7(Fig. 7) shows the FTIR profile of outer peel extracts (Figure 7a(Fig. 7)), inner peel extracts (Figure 7b(Fig. 7)), and juice (Figure 7c(Fig. 7)) of *P. granatum*. The appearance of broad absorbance at around 3421 cm^-1^, 3419 cm^-1^, and 3425 cm^-1 ^in the extracts of outer peel, inner peel, and juices respectively indicates the appearance of the -OH group of the phenolic and carboxylic group. The absorbance at 1726 cm^-1 ^and 1728 cm^-1^ indicates the presence of C=O (aldehyde/ketone) group in the extracts of outer and inner peels respectively, whereas it was absent in the juice extract. The presence of an aromatic group was observed in all the samples. Moreover, the FTIR results further support the result of GC-MS and encourages approach towards quantitative and qualitative analysis.

### In vitro antioxidant activity of PGEs

**Figure 8:**
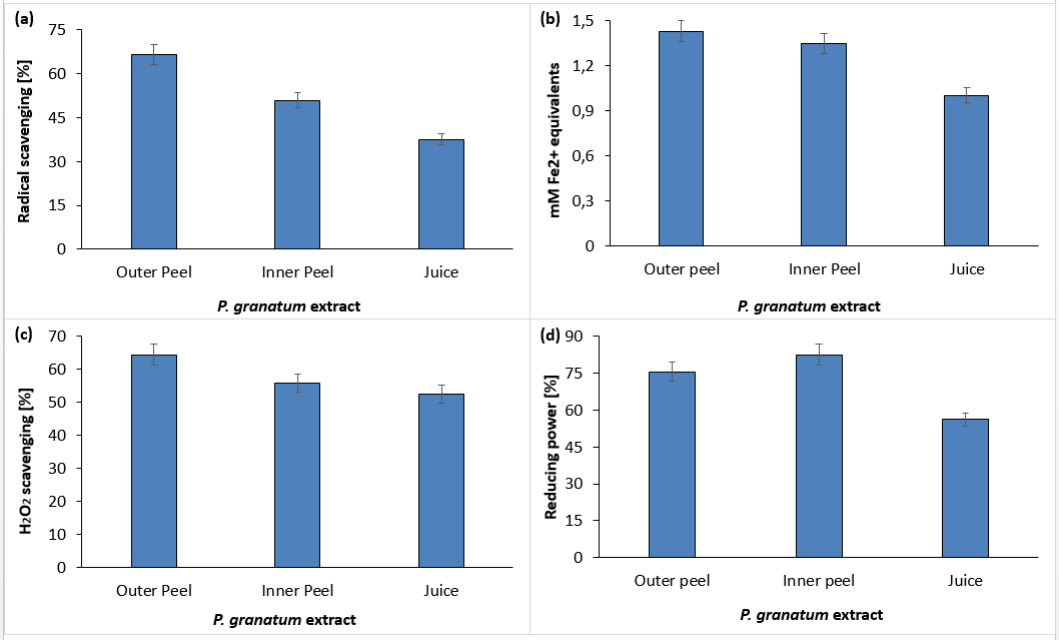
*In vitro* free radical scavenging activity of *P. granatum *by means of DPPH assay (a), FRAP assay (b), H_2_O_2_ scavenging assay (c), and reducing power assay (d).

The phytochemical constituents of pomegranate exhibited significant antioxidant activity (Gundogdu and Yilmaz, 2012[21]; Hmid et al., 2018[25]). Figure 8(Fig. 8) shows the total antioxidant function of PGEs, which were determined by DPPH (Figure 8a(Fig. 8)), FRAP (Figure 8b(Fig. 8)), Hydrogen Peroxide radical scavenging (Figure 8c(Fig. 8)) and reducing power assays (Figure 8d(Fig. 8)). The decolorization of DPPH was measured using a spectrophotometer. The DPPH scavenging activity of inner peel extract, outer peel extract, and fruit juice was found to be 50.89 %, 66.51 % and 37.5 % respectively (Figure 8a(Fig. 8)) (Supplementary Table 4).

The FRAP assay was also carried out to confirm the antioxidant properties of PGEs. In FRAP assay the potential of compounds to reduce ferric ion (Fe^3+^) to ferrous ion (Fe^2+^) was measured by determining the electron-donating ability of the test compound. Figure 8b(Fig. 8) shows the reducing ability of PGEs. All the parts of pomegranate exhibited higher reducing potential, however, the outer peel extract showed the inflated ferric reducing power (Supplementary Table 5). The FRAP assay is usually used to ensure the overall antioxidant potential of diverse natural products (Guo et al., 2003[22]; Rastogi et al., 2016[38]; Zaouay et al., 2012[49]).

The ability of *P. granatum *methanolic extract of outer peel, inner peel and juice to scavenge H_2_O_2_ was also assessed and percentage scavenging was found to be 64.50 %, 55.80 % and 52.55 % respectively (Figure 8c(Fig. 8)) (Supplementary Table 6). The antioxidant activity was concomitant with reducing power of plant materials. The more stable non-reactive species were formed by breaking the free radical chains in reducing power assay by donating H atoms indicated the presence of reductones (higher reducing power). In this study, the methanolic extract of *P. granatum* peels and juice showed its potent reducing power as shown in Figure 8d(Fig. 8). The increase in the absorbance at 700 nm indicates the better reducing power of test materials. Contrary to juice, the methanolic extract of inner and outer peels of *P. granatum* showed higher reducing power (Supplementary Table 7). Moreover, the antioxidant activity of PGEs was in agreement with previous studies (Derakhshan et al., 2018[18]; Gil et al., 2000[20]; Mphahlele et al., 2016[33]; Tezcan et al., 2009[45]).

### Life span assay 

**Figure 9:**
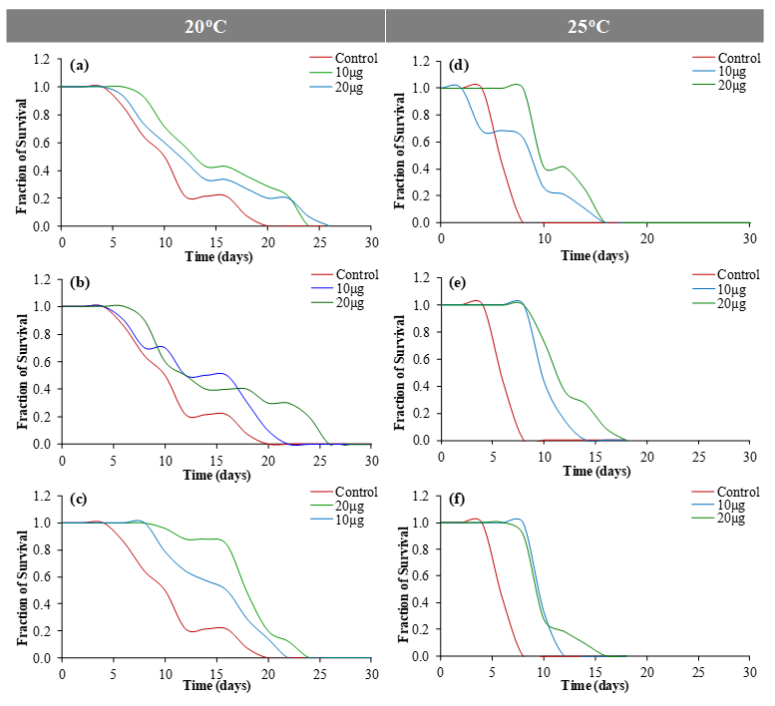
Lifespan assay using *C. elegans* at 20 °C [(a) outer peel; (b) inner peel; (c) juice] and 25 °C [(d) outer peel; (e) inner peel; (f) juice].

The antioxidant properties of PGEs were further explored as an active anti-aging compound using *C. elegans* as an eukaryotic model (Sonani et al., 2015[41]). The L4 stage (N2 Bristol) of* C. elegans *was cultured on NGM plates with and without methanolic PGEs of two different concentrations. Figure 9(Fig. 9) shows the percentage survival of the worms at 20 °C and 25 °C under PGEs of outer peels, inner peels and fruit juice. In comparison to the control sample, a remarkable elevation in the survival of the worms treated with PGEs was observed both at 20 °C and 25 °C. An increased life span of worms from 22 days (control) and 10 days (control) to 25 ± 1 days and 15 ± 1 days was observed at 20 °C and 25 °C respectively. Moreover, the worms supplemented with 20 µg/ml PGEs extract showed a significant increase in their lifespan in comparison to control samples. Interestingly, the PGEs of outer peels, inner peels and juice showed an almost similar increase in lifespan in a dose-dependent manner (Figure 9(Fig. 9)) (Supplementary Tables 8, 9, and 10). It has also been reported that PGEs showed an increase in lifespan and decrease fat deposition (intestine) in *C. elegans *(Zheng et al., 2017[53]). The PGEs (5 mg/ml) were found to advance the lifespan, formation of new generations, the productiveness of new generations, and the growth properties of *C. elegans *(Kiliçgün et al., 2015[30]).

### Survival of C. elegans under oxidative stress

**Figure 10:**
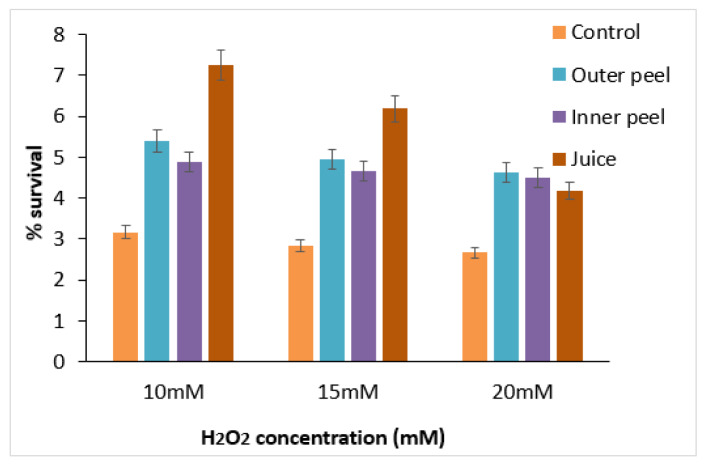
Survival of *C. elegans* treated with PGEs at different concentrations (10, 15, and 20 mM) of H_2_O_2_.

The oxidative stress tolerance of PGE (20 µg/ml) treated and untreated (control) worms were observed at different concentrations (10 mM, 15 mM, and 20 mM) of H_2_O_2 _(Figure 10(Fig. 10)). A decrease in percentage survival of the worms with increased concentration of H_2_O_2_ was observed, signifying the negative impacts of oxidative stress induced by the strong oxidizing agent. Moreover, in comparison to the control samples, a significant percentage increase in the life span of worms treated with PGEs of outer peels, inner peels and juice was observed. In comparison to inner peel extract and juice, the outer peel showed higher potential for reducing the oxidative stress to a certain extent, perhaps due to the presence of the comparatively higher content of polyphenols in the outer peels of pomegranate (Supplementary Table 11). The effect of pomegranate outer covering/peel (PRE) on oxidative stress was also studied, and it was observed that PRE may play an important role in preserving mitochondrial function during oxidative stress and attenuates paraquat toxicity (Mowery et al., 2018[32]).

### PGEs outer peel and inner peel effect on DAF-16

**Figure 11:**
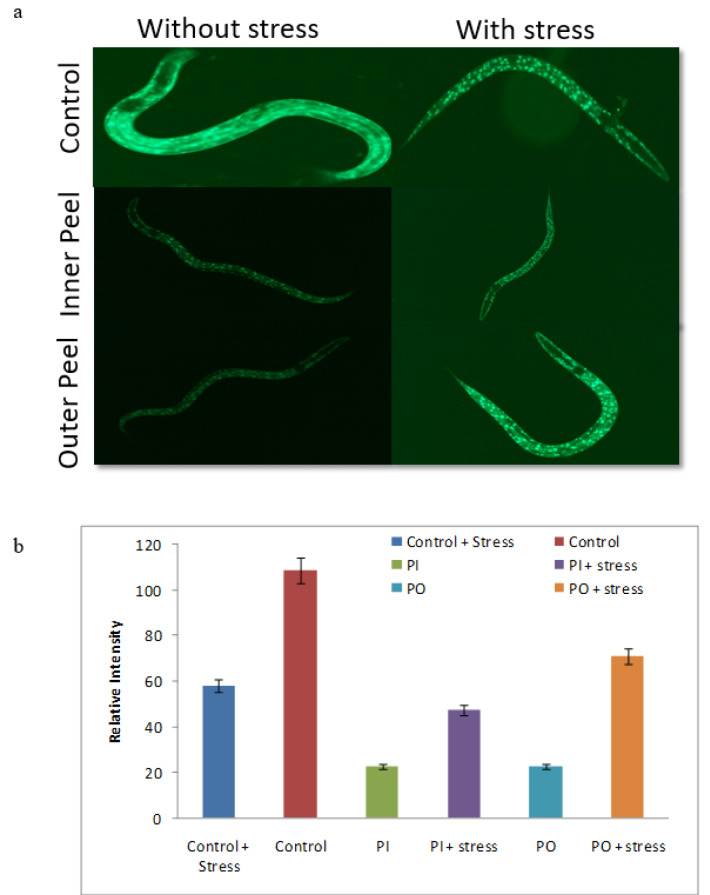
*DAF-16: GFP* nuclear localization assay (a) Nuclear translocation of DAF-16 in control, inner peel and outer peel incubated with thermal stress and without thermal stress and (b) Quantification of image intensity was done by Image J software.

The *DAF-16* transcription factor plays a key role in influencing the insulin signaling pathway. The Insulin growth factor-1 (IGF-1) and daf-2/age-1 inhibits the movement of *DAF-16 *from the cytoplasm to nuclei (Zečić and Braeckman, 2020[51]). The alteration in the activity of age-1 and daf-2 resulted in the expression of DAF-16 and consequently leads to shifting of* DAF-16 *to the nucleus which modulates the expression (upregulation) of anti-stress genes and longevity gene in the nuclei (Zhuang et al., 2014[55]). To check the result of PGEs outer peel and inner peel extract on the IGF-1 signaling pathway, we measured the appearance of the *DAF-16 *fork-head transcription factor in the cytoplasm of TJ356 which were translocated to the nucleus upon PGEs outer peel and inner peel exposure. It is extensively believed that *DAF-16 *restoration in the cytoplasm and its movement from cytoplasm to nucleus is an important indicator of an elevated level of longevity (dependent or independent on *DAF-16*). The level of *DAF16: *GFP intensity was declined in the PGEs supplemented worms (Figure 11(Fig. 11)). Consequently, positive rational can be proposed that upon PGEs supplementation to the worms, a significant reduction of *DAF-16* in the cytoplasm was seen, therefore it might be reliant on the *DAF-16 *fork-head transcription factor straight forward to increase the lifespan of worms. However, the exact mechanism of how it works is yet unrevealed.

## Conclusion

In this study, pomegranate's outer/inner peels and juice were analyzed for the occurrence of total phenolic compounds along with their antioxidant function and anti-aging benefits. The PGEs comprise a sizeable quantity of phenolic compounds with high antioxidant/free radical scavenging activity. It was noticed that the outer peel contains elevated constituents of overall phenols and also contributed to higher free radical scavenging activity, while the inner peel and juice were rich in flavonoids and vitamin C, respectively. The lifespan assays showed that PGEs may significantly extend the mean lifespan of *C. elegans* by reducing the oxidative stress generated by hydrogen peroxide. Outer peel showed higher anti-aging activity in comparison with inner peel and juice. Additionally, the result of PGEs outer peel and inner peel exposure to the TJ356 worms confirmed the *DAF-16 *dependent elevated longevity in worms. However, exact mechanisms exerted by a particular bioactive compound responsible for the longevity of *C. elegans* are yet to be verified. The current findings indicate that due to high antioxidant and anti-aging capacity, pomegranate could be used as a supplement to enhance the growth and survival rate. The pomegranate extracts can be used as a natural pharmaceutical product for treating neurodegenerative diseases and oxidative stress-induced disorders.

## Notes

Mukesh G. Chaubey and Anita P. Chauhan contributed equally as first authors.

Rajesh P. Rastogi and Niraj Kumar Singh (Department of Biotechnology, Shri Alpesh N. Patel P.G. Institute of Science and Research, Anand-388001, Gujarat, India; E-mail: nirajbiotech@gmail.com) contributed equally as corresponding authors.

**
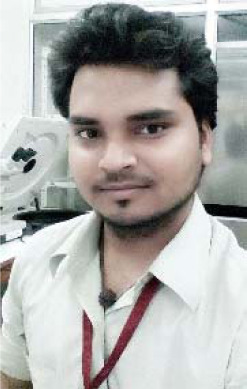
**

**Humble tribute to Mr. Mukesh G. Chaubey †**

Mr. Mukesh G. Chaubey was born on 20^th^ May, 1991 at Varanasi, India. He was pursuing Ph.D. in Biotechnology from Department of Biotechnology, Shri A. N. Patel P. G. Institute of Science and Research, Anand, Gujarat, India. Recently, he had submitted his Ph.D. thesis on 23^rd^ October, 2020. Unfortunately, Mr. Mukesh passed away on 23^rd^ November, 2020 due to Covid-19. We have lost one of our sincere and young colleague. He will always remain within our hearts. We pray that his soul rest in peace and may God give enough strength to the bereaved family to bear the irreparable loss. We don't have any words to express our grief. This publication is sincerely dedicated to our young colleague who sadly left us too early.

## Acknowledgement

NKS acknowledge Science and engineering research board (SERB), Department of Science and Technology (DST), Ministry of Science and Technology, Government of India, for Young Scientist fellowship under the scheme of a startup research grant (SB/YS/LS-290/2013). MGC also acknowledge SERB for Senior Research fellowship under the Scheme of a startup research grant (SB/YS/LS-290/2013). The authors also acknowledge SICART supported by the Department of Science and Technology, managed by Charutar Vidya Mandal, Vallabh Vidhyanagar, Anand for GC-MS and FTIR facility.

## Conflicts of interest

The authors have no conflict of interest to declare.

## Supplementary Material

Supplementary data

## Figures and Tables

**Table 1:**
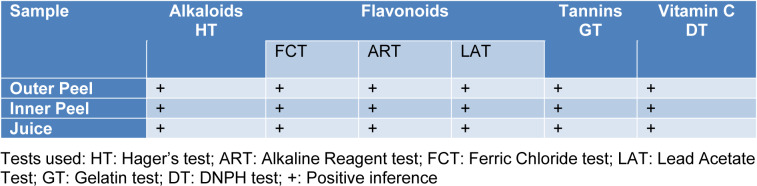
The spot tests of *Punica granatum* were performed for investigating the presence of alkaloids, flavonoids, tannins, and vitamin C

**Table 2:**
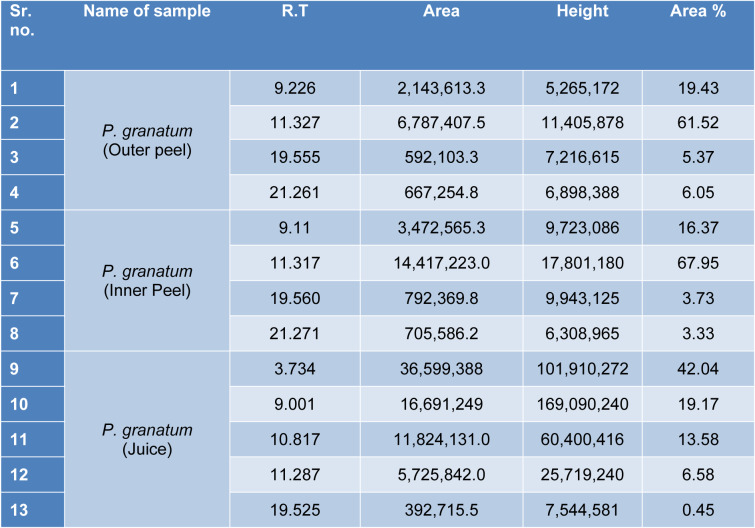
The *P. granatum *extracts were analyzed through gas chromatography and the percentage of the total area of the peaks of the GC spectrum of some active compounds is mentioned.

**Table 3:**
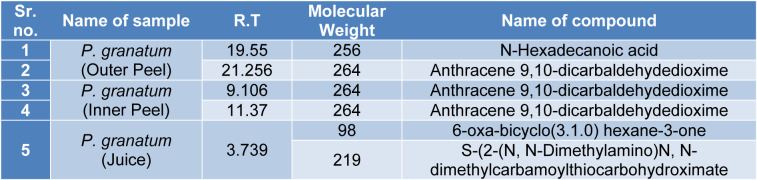
After GC analysis *P. granatum* extracts were examined by mass spectrometry and the table shows the presence of some major phytoconstituents in different methanolic extracts.

**Table 4:**
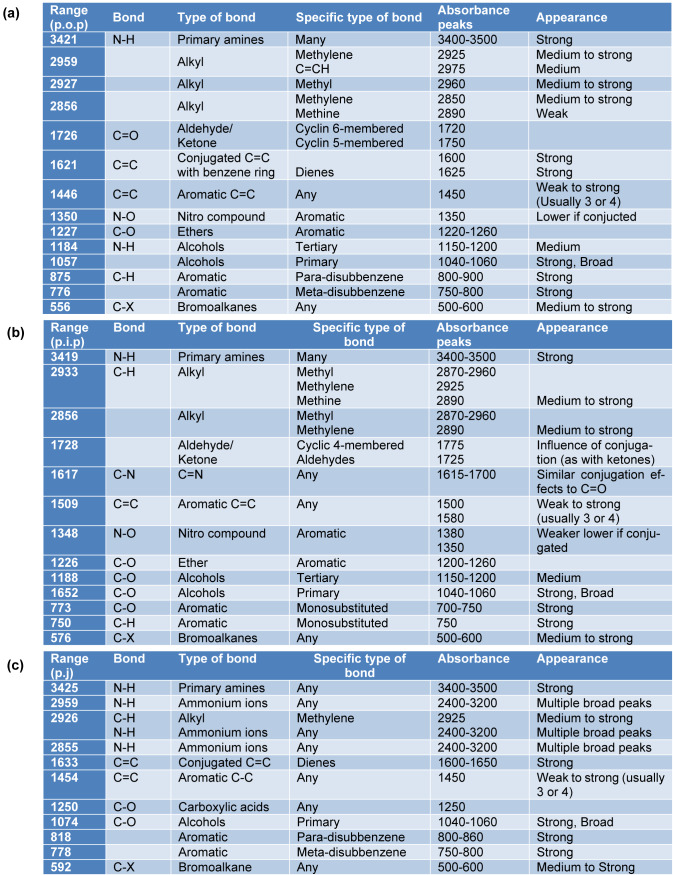
The FTIR spectrum of the *P. granatum *extracts showed the major absorbance bands in each of the samples (a) *P. granatum *outer peel (p.o.p), (b) *P. granatum *inner peel (p.i.p), and (c) *P. granatum *juice (p.j).

**Figure 1:**
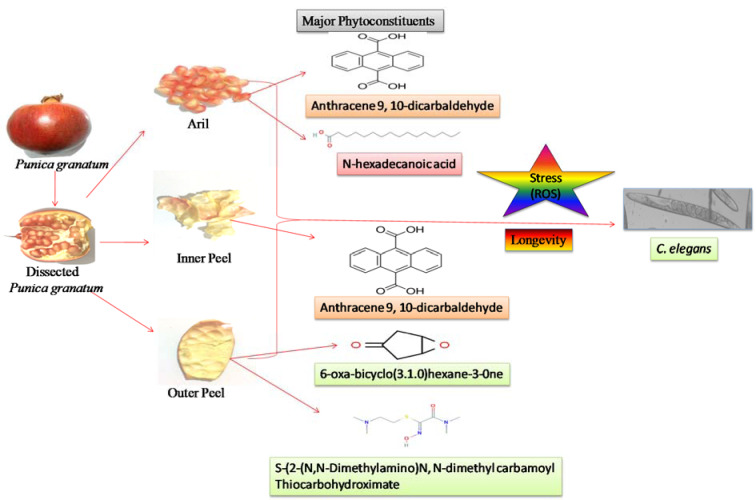
Graphical abstract
