# Supplementary material for: Therapeutic potential of bioactive compounds from Punica granatum extracts against aging and complicity of FOXO orthologue DAF-16 in Caenorhabditis elegans
Source: EXCLI J. 2021 Jan 11;20:80–98. doi: 10.17179/excli2020-3011 (PMC7838930; doi:10.17179/excli2020-3011)
Supplement: Supplementary data [file EXCLI-20-80-s-001.pdf]

EXCLI Journal 2021;20:80-98 – ISSN 1611-2156
Received: October 13, 2020, accepted: January 04, 2021, published: January 11, 2021

Supplementary data to:

Original article:

THERAPEUTIC POTENTIAL OF BIOACTIVE COMPOUNDS FROM *PUNICA GRANATUM* EXTRACTS AGAINST AGING AND COMPLICITY OF FOXO ORTHOLOGUE DAF-16 IN *CAENORHABDITIS ELEGANS*

Mukesh G. Chaubey[1,#,†], Anita P. Chauhan[1#], Pooja R. Chokshi[1], Rahi S. Amin[1], Stuti N. Patel[2], Datta Madamwar[3], Rajesh P. Rastogi[4]*, Niraj Kumar Singh[1]*

[1] Department of Biotechnology, Shri Alpesh N. Patel P.G. Institute of Science and Research, Anand-388001, Gujarat, India
[2] Post-Graduate Department of Biosciences, UGC-Centre of Advanced Study, Sardar Patel University, Vadtal Road, Satellite Campus, Bakrol, Anand, Gujarat 388 315, India
[3] P. D. Patel Institute of Applied Sciences, Charotar University of Science and Technology, CHARUSAT campus, Changa 388421, Anand, Gujarat, India
[4] Ministry of Environment, Forest and Climate Change, Indira Paryavaran Bhawan, New Delhi 110003, India

[#] Authors contributed equally

* **Corresponding authors:**
Rajesh P Rastogi, Ministry of Environment, Forest and Climate Change, Indira Paryavaran Bhawan, New Delhi 110003, India; E-mail: raj_rastogi@rediffmail.com
Niraj Kumar Singh, Department of Biotechnology, Shri Alpesh N. Patel P.G. Institute of Science and Research, Anand-388001, Gujarat, India; E-mail: nirajbiotech@gmail.com

https://orcid.org/0000-0001-8519-3582 (Mukesh G Chaubey)
https://orcid.org/0000-0002-9588-1167 (Anita P. Chauhan)
https://orcid.org/0000-0002-8290-9669 (Pooja R. Chokshi)
https://orcid.org/0000-0002-7927-7779 (Rahi S. Amin)
https://orcid.org/0000-0002-2445-2680 (Stuti N. Patel)
https://orcid.org/0000-0003-3301-1120 (Datta Madamwar)
https://orcid.org/0000-0003-1500-7371 (Rajesh P. Rastogi)
https://orcid.org/0000-0002-2365-2314 (Niraj Kumar Singh)

http://dx.doi.org/10.17179/excli2020-3011

This is an Open Access article distributed under the terms of the Creative Commons Attribution License (http://creativecommons.org/licenses/by/4.0/).

EXCLI Journal 2021;20:80-98 – ISSN 1611-2156
Received: October 13, 2020, accepted: January 04, 2021, published: January 11, 2021

Supplementary Table 1: A) The standard curve of tannic acid was prepared to obtain the total phenol content (TPC) from samples; **B)** The OD of replicates for all the samples, its average, standard deviation, and the TPC obtained are mentioned. (Raw data to Figure 3a in the main document)

A)

**Aliquots conc. (µg)**	**OD at 760 nm**				
	**1**	**2**	**3**	**AVG**	**SD**
40	0.165	0.156	0.162	0.161	0.004583
80	0.227	0.218	0.221	0.222	0.004583
120	0.398	0.386	0.389	0.391	0.006245
160	0.464	0.46	0.462	0.462	0.002
200	0.54	0.547	0.548	0.545	0.004359
240	0.61	0.62	0.615	0.615	0.005

1, 2, 3: Replicate number; OD: Optical density; AVG: Average; SD: Standard Deviation
Standard Tannic Acid (1 mg/ml)
Sample (1 mg/ml) and volume (50 µl)
Standard curve equation obtained was: R^2=0.978; Y=0.0024X + 0.0683

B)

**Samples**	**OD of replicates**			**AVG**	**SD**	**TPC (mg TAE/gm of extract)**
	**1**	**2**	**3**			
Outer peel	0.516	0.527	0.534	0.525666667	0.009073772	190.55
Inner peel	0.088	0.095	0.082	0.088333333	0.006506407	8.33
Juice	0.137	0.128	0.14	0.135	0.006244998	27.79

1, 2, 3: Replicate number; OD: Optical density; AVG: Average; SD: Standard Deviation
The total phenol content (TPC) was obtained using the following equation:
TPC=C×V/W
Where, C= Conc. From std. curve (mg/ml)
V= Vol. of sample used in experiment in ml
W= Weight of sample in gm

EXCLI Journal 2021;20:80-98 – ISSN 1611-2156
Received: October 13, 2020, accepted: January 04, 2021, published: January 11, 2021

Supplementary Table 2: A) The standard curve of quercetin was prepared to obtain the total flavonoid content (TFC) from samples; **B)** The OD of replicates for all the samples, its average, standard deviation, and the TFC obtained are mentioned. (Raw data to Figure 3b in the main document)

A)

Aliquots conc. (µg)	OD at 510 nm				
	1	2	3	AVG	SD
10	0.143	0.146	0.149	0.146	0.003
20	0.185	0.178	0.18	0.181	0.003606
30	0.221	0.224	0.212	0.219	0.006245
40	0.281	0.28	0.276	0.279	0.002646
50	0.304	0.306	0.296	0.302	0.005292
60	0.347	0.339	0.349	0.345	0.005292
70	0.379	0.378	0.374	0.377	0.002646
80	0.442	0.444	0.437	0.441	0.003606
90	0.491	0.495	0.487	0.491	0.004
100	0.537	0.532	0.539	0.536	0.003606

1, 2, 3: Replicate number; OD: Optical density; AVG: Average; SD: Standard Deviation
Standard Quercetin (1 mg/ml)
Sample (1 mg/ml) and volume (30 µl)
Standard curve equation obtained was: $R^2=0.995$; $Y=0.0043X + 0.0941$

B)

Samples	OD of replicates			AVG	SD	(TFC) mg QE/gm of extract
	1	2	3			
Outer peel	0.1051	0.1101	0.0982	0.104466667	0.0059752	2.39
Inner peel	0.122	0.1131	0.109	0.1147	0.0066461	4.8
Juice	0.089	0.1022	0.0931	0.094766667	0.006756	0.154

1, 2, 3: Replicate number; OD: Optical density; AVG: Average; SD: Standard Deviation
The total flavonoid content (TFC) was obtained using the following equation:
TFC=C×V/W
Where, C= Conc. From std. curve (mg/ml)
V= Vol. of sample used in experiment in ml
W= Weight of sample in gm

EXCLI Journal 2021;20:80-98 – ISSN 1611-2156
Received: October 13, 2020, accepted: January 04, 2021, published: January 11, 2021

Supplementary Table 3: A) The standard curve of quercetin was prepared to obtain the total vitamin C content (TVC) from samples; **B)** The OD of replicates for all the samples, its average, standard deviation, and the TVC obtained are mentioned. (Raw data to Figure 3c in the main document)

A)

Aliquots conc. (µg)	OD at 540 nm				
	1	2	3	AVG	SD
Blank	0	0	0	0	0
10	0.007	0.009	0.005	0.007	0.002
20	0.08	0.12	0.07	0.09	0.026458
30	0.131	0.133	0.126	0.13	0.003606
40	0.152	0.151	0.147	0.15	0.002646
50	0.179	0.185	0.176	0.18	0.004583
60	0.221	0.224	0.215	0.22	0.004583
70	0.229	0.234	0.227	0.23	0.003606
80	0.252	0.252	0.246	0.25	0.003464

1, 2, 3: Replicate number; OD: Optical density; AVG: Average; SD: Standard Deviation
Standard Ascorbic acid (1 mg/ml)
Sample (1 mg/ml) and volume (200 µl)
Standard curve equation obtained was: $R^2=0.933$; $Y=0.0034X$

B)

Samples	OD of replicates			AVG	SD	TVC (mg AAE/gm of extract)
	1	2	3			
Outer peel	0.00181	0.00196	0.00189	0.001886667	7.5056E-05	0.555
Inner peel	0.0041	0.005	0.0037	0.004266667	0.00066583	1.25
Juice	0.0051	0.0062	0.0059	0.005733333	0.00056862	1.685

1, 2, 3: Replicate number; OD: Optical density; AVG: Average; SD: Standard Deviation
The total vitamin C content (TVC) was obtained using the following equation:
$TVC=C \times V/W$
Where, C= Conc. From std. curve (mg/ml)
V= Vol. of sample used in experiment in ml
W= Weight of sample in gm

EXCLI Journal 2021;20:80-98 – ISSN 1611-2156
Received: October 13, 2020, accepted: January 04, 2021, published: January 11, 2021

Supplementary Table 4: The antioxidant activity of outer peel, inner peel and juice analyzed by *in vitro* DPPH scavenging assay. (Raw data to Figure 8a in the main document)

**Samples**	**OD of replicates**			**AVG**	**SD**	**% Inhibition**
	**1**	**2**	**3**			
Outer Peel	0.076	0.073	0.077	0.075333	0.002082	66.51
Inner Peel	0.117	0.105	0.11	0.110667	0.006028	50.89
Juice	0.135	0.145	0.14	0.14	0.005	37.5

1, 2, 3: Replicate number

Supplementary Table 5: A) Standard curve of $FeSO_4$ to obtain the FRAP value; **B)** The antioxidant activity of outer peel, inner peel and juice analyzed by *in vitro* Ferric Reducing Antioxidant Power Assay (FRAP assay). (Raw data to Figure 8b in the main document)

A)

**Aliquots conc. (µM)**	**OD at 593 nm**			**AVG**	**SD**
	**1**	**2**	**3**		
100	0.076	0.071	0.078	0.075	0.003606
200	0.142	0.145	0.133	0.14	0.006245
300	0.209	0.213	0.208	0.21	0.002646
400	0.267	0.26	0.262	0.263	0.003606
500	0.313	0.316	0.304	0.311	0.006245
600	0.37	0.369	0.365	0.368	0.002646
700	0.418	0.411	0.419	0.416	0.004359
800	0.496	0.498	0.488	0.494	0.005292
900	0.512	0.516	0.505	0.511	0.005568
1000	0.56	0.561	0.568	0.563	0.004359

1, 2, 3: Replicate number
Standard curve equation obtained was: $R^2=0.981$; $Y=0.0006X$

B)

**Samples**	**OD of replicates**			**AVG**	**SD**	**mM Fe^{2+} equivalents**
	1	2	3			
Outer peel	0.867	0.854	0.859	0.86	0.006557	1.433
Inner Peel	0.808	0.817	0.805	0.81	0.006245	1.35
Juice	0.602	0.61	0.6	0.604	0.005292	1.006

1, 2, 3: Replicate number

EXCLI Journal 2021;20:80-98 – ISSN 1611-2156
Received: October 13, 2020, accepted: January 04, 2021, published: January 11, 2021

Supplementary Table 6: The antioxidant activity of outer peel, inner peel and juice analyzed by *in vitro* H_2O_2 scavenging assay. (Raw data to Figure 8c in the main document)

**Samples**	**OD of replicates**			**AVG**	**SD**	**Percentage scavenging of H_2O_2 (%)**
	**1**	**2**	**3**			
Control	0.1174	0.1025	0.1317	0.1172	0.014601	-
Outer peel	0.03953	0.04377	0.04164	0.041647	0.00212	64.5
Inner Peel	0.06288	0.04182	0.0508	0.051833	0.010568	55.8
Juice	0.05032	0.060702	0.055814	0.055612	0.005194	52.55

1, 2, 3: Replicate number

Supplementary Table 7: The antioxidant activity of outer peel, inner peel and juice analyzed by *in vitro* **R**educing Power Assay. (Raw data to Figure 8d in the main document)

**Samples**	**OD of replicates**			**AVG**	**SD**	**% Increase in RPA**
	1	2	3			
Control	0.318	0.323	0.31	0.317	0.006557439	-
Outer peel	0.562	0.552	0.557	0.557	0.005	75.7
Inner peel	0.59	0.569	0.579	0.579333	0.010503968	82.64
Juice	0.476	0.514	0.495	0.495	0.019	56.15

1, 2, 3: Replicate number

EXCLI Journal 2021;20:80-98 – ISSN 1611-2156
Received: October 13, 2020, accepted: January 04, 2021, published: January 11, 2021

Supplementary Table 8: Number of dead worms over a time period of days maintained at **A)** 20 °C and **B)** 25 °C under the treatment of outer peel of *P. granatum*, and the fraction of survival was calculated using prism software. (**A**) Raw data to Figure 9a and **B)** Figure 9d in the main document)

A)

Days	At 20 °C														
	Control					10 µg					20 µg				
	1	2	3	AVG	Fraction of survival	1	2	3	AVG	Fraction of survival	1	2	3	AVG	Fraction of survival
0	0	0	0	0	1	0	0	0	0	1	0	0	0	0	1
2	0	0	0	0	1	0	0	0	0	1	0	0	0	0	1
4	0	0	0	0	1	0	0	0	0	1	0	0	0	0	1
6	3	2	1	2	0.857143	0	0	0	0	1	2	0	1	1	0.933333
8	3	2	4	3	0.642857	0	2	1	1	0.928571	3	4	2	3	0.733333
10	1	3	2	2	0.5	4	2	3	3	0.714286	2	3	1	2	0.6
12	3	5	4	4	0.214286	1	2	3	2	0.571429	2	1	3	2	0.466667
14	0	0	0	0	0.214286	2	3	1	2	0.428571	2	1	3	2	0.333333
16	0	0	0	0	0.214286	0	0	0	0	0.428571	0	0	0	0	0.333333
18	1	2	3	2	0.071429	1	0	2	1	0.357143	0	1	2	1	0.266667
20	1	2	0	1	0	2	1	0	1	0.285714	2	0	1	1	0.2
22	0	0	0	0	0	2	0	1	1	0.214286	0	0	0	0	0.2
24						3	4	2	3	0	2	1	3	2	0.066667
26						0	0	0	0	0	1	0	2	1	0
28											0	0	0	0	0

1, 2, 3: Replicate number; AVG: Average

B)

Days	At 25 °C														
	Control					10 µg					20 µg				
	1	2	3	AVG	Fraction of survival	1	2	3	AVG	Fraction of survival	1	2	3	AVG	Fraction of survival
0	0	0	0	0	1	0	0	0	0	1	0	0	0	0	1
2	0	0	0	0	1	0	0	0	0	1	0	0	0	0	1
4	0	0	0	0	1	5	6	7	6	0.684211	0	0	0	0	1
6	3	5	4	4	0.333333333	0	0	0	0	0.684211	0	0	0	0	1
8	3	1	2	2	0	2	0	1	1	0.631579	0	0	0	0	1
10	0	0	0	0	0	8	6	7	7	0.263158	6	7	8	7	0.416666667
12						0	2	1	1	0.210526	0	0	0	0	0.416666667
14						2	1	3	2	0.105263	2	1	3	2	0.25
16						3	1	2	2	0	3	2	4	3	0
18						0	0	0	0	0	0	0	0	0	0

1, 2, 3: Replicate number; AVG: Average

EXCLI Journal 2021;20:80-98 – ISSN 1611-2156
Received: October 13, 2020, accepted: January 04, 2021, published: January 11, 2021

Supplementary Table 9: Number of dead worms over a time period of days maintained at **A.** 20°C and **B.** 25°C under the treatment of inner peel of *P. granatum*, and the fraction of survival was calculated using prism software. (**A**) Raw data to Figure 9b and **B)** Figure 9e in the main document)

A)

Days	At 20 °C														
	Control					10 µg					20 µg				
	1	2	3	AVG	Fraction of survival	1	2	3	AVG	Fraction of sur-vival	1	2	3	AVG	Fraction of sur-vival
**0**	0	0	0	0	1	0	0	0	0	1	0	0	0	0	1
**2**	0	0	0	0	1	0	0	0	0	1	0	0	0	0	1
**4**	0	0	0	0	1	0	0	0	0	1	0	0	0	0	1
**6**	2	2	2	2	0.857143	1	0	2	1	0.9	0	0	0	0	1
**8**	2	3	4	3	0.642857	3	2	1	2	0.7	2	0	1	1	0.9
**10**	4	1	1	2	0.5	0	0	0	0	0.7	4	3	2	3	0.6
**12**	5	3	4	4	0.214286	2	1	3	2	0.5	1	0	2	1	0.5
**14**	0	0	0	0	0.214286	0	0	0	0	0.5	0	2	1	1	0.4
**16**	0	0	0	0	0.214286	0	0	0	0	0.5	0	0	0	0	0.4
**18**	2	2	2	2	0.071429	2	3	1	2	0.3	0	0	0	0	0.4
**20**	0	1	2	1	0	2	1	3	2	0.1	1	0	2	1	0.3
**22**	0	0	0	0	0	1	0	2	1	0	0	0	0	0	0.3
**24**						0	0	0	0	0	1	0	2	1	0.2
**26**											2	1	3	2	0
**28**											0	0	0	0	0

1, 2, 3: Replicate number; AVG: Average

B)

Days	At 25 °C														
	Control					10 µg					20 µg				
	1	2	3	AVG	Fraction of sur-vival	1	2	3	AVG	Fraction of sur-vival	1	2	3	AVG	Fraction of sur-vival
**0**	0	0	0	0	1	0	0	0	0	1	0	0	0	0	1
**2**	0	0	0	0	1	0	0	0	0	1	0	0	0	0	1
**4**	0	0	0	0	1	0	0	0	0	1	0	0	0	0	1
**6**	5	3	4	4	0.428571	0	0	0	0	1	0	0	0	0	1
**8**	4	3	2	3	0	0	0	0	0	1	0	0	0	0	1
**10**	0	0	0	0	0	3	4	5	4	0.428571	2	3	4	3	0.727273
**12**						3	2	1	2	0.142857	3	5	4	4	0.363636
**14**						0	2	1	1	0	2	0	1	1	0.272727
**16**						0	0	0	0	0	1	3	2	2	0.090909
**18**											1	0	2	1	0
**20**											0	0	0	0	0

1, 2, 3: Replicate number; AVG: Average

EXCLI Journal 2021;20:80-98 – ISSN 1611-2156
Received: October 13, 2020, accepted: January 04, 2021, published: January 11, 2021

Supplementary Table 10: Number of dead worms over a time period of days maintained at **A)** 20 °C and **B)** 25 °C under the treatment of juice of *P. granatum*, and the fraction of survival was calculated using prism software. (**A**) Raw data to Figure 9c and **B)** Figure 9f in the main document)

A)

Days	At 20 °C														
	Control					10 µg					20 µg				
	1	2	3	AVG	Fraction of survival	1	2	3	AVG	Frac-tion of sur-vival	1	2	3	AVG	Fraction of survival
**0**	0	0	0	0	1	0	0	0	0	1	0	0	0	0	1
**2**	0	0	0	0	1	0	0	0	0	1	0	0	0	0	1
**4**	0	0	0	0	1	0	0	0	0	1	0	0	0	0	1
**6**	2	3	1	2	0.857143	0	0	0	0	1	0	0	0	0	1
**8**	3	2	4	3	0.642857	0	0	0	0	1	0	0	0	0	1
**10**	1	3	2	2	0.5	0	2	1	1	0.96	4	2	3	3	0.785714
**12**	4	3	5	4	0.214286	2	1	3	2	0.88	1	4	1	2	0.642857
**14**	0	0	0	0	0.214286	0	0	0	0	0.88	1	2	0	1	0.571429
**16**	0	0	0	0	0.214286	1	2	0	1	0.84	2	0	1	1	0.5
**18**	3	1	2	2	0.071429	1	8	8	9	0.48	4	2	3	3	0.285714
**20**	2	1	0	1	0	6	9	6	7	0.2	1	2	3	2	0.142857
**22**	0	0	0	0	0	2	2	2	2	0.12	2	2	2	2	0
**24**						4	3	2	3	0	0	0	0	0	0
**26**						0	0	0	0	0					

1, 2, 3: Replicate number; AVG: Average.

B)

Days	At 25 °C														
	Control					10 µg					20 µg				
	1	2	3	AVG	Fraction of survival	1	2	3	AVG	Fraction of survival	1	2	3	AVG	Fraction of survival
**0**	0	0	0	0	1	0	0	0	0	1	0	0	0	0	1
**2**	0	0	0	0	1	0	0	0	0	1	0	0	0	0	1
**4**	0	0	0	0	1	0	0	0	0	1	0	0	0	0	1
**6**	4	5	3	4	0.42857142	0	0	0	0	1	0	0	0	0	1
**8**	2	4	3	3	0	0	0	0	0	1	0	1	2	1	0.90909090
**10**	0	0	0	0	0	2	4	6	4	0.33333333	8	7	6	7	0.27272727
**12**						1	3	2	2	0	3	0	0	1	0.18181818
**14**						0	0	0	0	0	0	2	1	1	0.09090909
**16**											0	1	2	1	0
**18**											0	0	0	0	0

1, 2, 3: Replicate number; AVG: Average.

EXCLI Journal 2021;20:80-98 – ISSN 1611-2156
Received: October 13, 2020, accepted: January 04, 2021, published: January 11, 2021

Supplementary Table 11: % survival of treated and control *C. elegans* under the oxidative stress produced by different concentrations of H_2O_2. (Raw data to Figure 10 in the main document)

**Conc. of H_2O_2**	**10 mM**				**15 mM**				**20 mM**			
	**C**	**OP**	**IP**	**J**	**C**	**OP**	**IP**	**J**	**C**	**OP**	**IP**	**J**
**1**	3.168	5.386	4.872	7.3	2.836	4.948	4.665	6.25	2.66	4.628	4.3	4.26
**2**	3.17	5.372	4.894	7.19	2.839	4.954	4.66	6.13	2.671	4.633	5	4.16
**3**	3.16	5.394	4.874	7.26	2.824	4.941	4.655	6.17	2.662	4.614	4.2	4.14
**AVG**	3.166	5.384	4.88	7.25	2.833	4.947	4.66	6.183	2.664	4.625	4.5	4.186
**SD**	0.005292	0.011136	0.012166	0.055678	0.007937	0.006506	0.005	0.061101	0.005859	0.009849	0.43589	0.064291

C: Control; OP: Outer Peel; IP: Inner Peel; J: Juice; AVG: Average; SD: Standard Deviation

1, 2, 3: replicate numbers
